# Assessment of Lactose-Free Diet on the Phalangeal Bone Mineral Status in Italian Adolescents Affected by Adult-Type Hypolactasia

**DOI:** 10.3390/nu10050558

**Published:** 2018-05-01

**Authors:** Alessandro Baldan, Sylvie Tagliati, Daniela Saccomandi, Andrea Brusaferro, Laura Busoli, Andrea Scala, Cristina Malaventura, Giuseppe Maggiore, Caterina Borgna-Pignatti

**Affiliations:** Department of Medical Sciences, Section of Pediatrics, University of Ferrara, Via A. Moro 8, 44124 Ferrara, Italy; alessandro.baldan@gmail.com (A.Ba.); snd@unife.it (D.S.); andrea.brusaferro@student.unife.it (A.Br.); busolilaura@gmail.com (L.B.); andrea.scala@student.unife.it (A.S.); mlvcst@unife.it (C.M.); mgggpp@unife.it (G.M.); bre@unife.it (C.B.-P.)

**Keywords:** phalangeal quantitative ultrasound, lactose intolerance, calcium intake, lactose-free milk

## Abstract

Adult-type hypolactasia (ATH) is a clinical syndrome of primary lactase deficiency. A lactose-free diet is advisable to avoid the symptoms linked to the condition, but this potentially creates problems for optimal bone mineralization due to reduced calcium intake. To evaluate the effect of the lactose-free diet on the bone mineral status (BMS), we compared the phalangeal BMS of adolescents with ATH to that of peers on a normal diet. Also, we analyzed the correlations between BMS and dietary behavior, physical exercise, and calcium and vitamin D intake. A total of 102 cases and 102 healthy controls filled out a diet record and underwent phalangeal Quantitative Ultrasound (QUS). No difference in BMS was observed. The time spent on lactose-free diet (4.8 ± 3.1 years) was inversely correlated to the BMS. More than 98% of cases consumed lactose-free milk, but calcium and vitamin D intake were significantly lower. Calcium intake was correlated to physical exercise but not to BMS. Our results suggest that a lactose-free diet does not affect the phalangeal BMS of adolescents with primary lactase deficiency when their diet includes lactose-free cow’s milk. However, there is still a significantly lower calcium intake than in the population reference. The inverse correlation observed between the BMS and the time spent on a lactose-free diet suggests that a long-term follow-up is advisable.

## 1. Introduction

The ability to digest lactose depends on the gene coding for lactase (lactase-phlorizin hydrolase, LCT; MIM 603202), an enzyme located in the brush border of the small intestine and localized to the tip of the villi [1]. Lactase concentrations are at their highest shortly after birth and decline rapidly after the usual age of weaning. Polymorphic variants in the lactase gene allow the digestion of milk and dairy products in adulthood, which is referred to lactose persistence (LP). LP became important in nomadic pastoral populations where the polymorphic variants differed among populations throughout Europe and part of Africa and the Middle East [2,3]. The inability to digest milk and dairy product is referred to as lactase deficiency and can be classified as primary, secondary, congenital, and developmental [4]. In particular, people with primary lactase deficiency have a physiological decline in lactase concentrations that occurs at the age of weaning. Adult-type hypolactasia (ATH), also known as lactase non-persistence or lactose intolerance (LI), is a clinical syndrome of primary lactase deficiency with one or more of the following: abdominal pain, diarrhea, nausea, flatulence, and bloating after the ingestion of lactose or lactose-containing food. The amount of lactose that causes the symptoms varies from individual to individual, depending on the amount of lactose consumed, the degree of lactase deficiency, and the form of food in which the lactose is ingested [5]. The American Academy of Pediatrics supports the use of dairy products as an important source of calcium (Ca) for bone mineral health and a correct growth in children and adolescents [5,6]. For subjects with LI, a lactose-free diet is usually prescribed to avoid symptoms linked to their condition. Consequently, it creates problems for optimal bone mineralization. In fact, adequate Ca intake during the growth period is critical for reaching optimal bone growth and maximum peak bone mass [7]. The most rapid skeletal development, which occurs in childhood and adolescence, would account for 30–40% of the total bone mass [8,9]. Along with Ca intake, an adequate 25-hydroxy vitamin D (indicated as vitamin D throughout the manuscript) intake is required for a normal bone mineral status. The bone mineral status (BMS) appears to be genetically determined up to 80%, whereas environmental factors, such as weight, physical exercise, and dietary intake of Ca and vitamin D, could affect it up to 20% [10].

Some studies reported that a reduced value of a quantitative ultrasound (QUS) variable, both velocity- and attenuation-based, was associated with a reduced BMS in children with growth problems or disorders affecting bone health [11,12,13,14]. One study examined the correlation between ATH and bone mineral density (BMD), but their subjects were also affected by cystic fibrosis [15], and the authors used dual-emission X-ray absorptiometry (DXA) instead of QUS. Our study also provides an overview of Ca intake from different foods that are a source of Ca and vitamin D.

The aim of this study was to evaluate the effect of a lactose-free diet on the BMS in primary-lactase-deficiency adolescents and compare it to a similar population on a diet without restriction (regular diet) using phalangeal QUS, a radiation-free technique. Furthermore, we looked for possible correlations between phalangeal BMS and diet, physical exercise, and Ca and vitamin D intake.

## 2. Materials and Methods

### 2.1. Subjects

The case group (lactose malabsorbers) was formed by 102 northeastern Italian adolescents (age 16.8 ± 2.6 years) of Caucasian ancestry, who had been in the past referred to the pediatric Gastroenterology clinic of the University Hospital S. Anna, Ferrara, Italy (Latitude 44°50′0″ N). All of them presented a positive hydrogen breath test and had a genotype compatible with primary lactase deficiency. The cases were further subdivided in lactose-intolerant (LI, *n* = 41) or lactose-tolerant (LT, *n* = 61) on the basis of whether or not they had experienced gastrointestinal symptoms during the breath test.

Some of these patients were from a previous study by our group directed to investigate the −13,910 C/T polymorphism for the adult-type genetic testing hypolactasia in a Southern European population [16]. At the time of this study, they were invited by telephone to return to the clinic for a check-up several months after diagnosis. They were also requested to fill out a diet record for the week preceding the day of the examination. During the present study, none of them were taking Ca, Vitamin D supplementation, or prescribed medications.

The control group was formed by 102 healthy volunteer teenagers from a local high school on a diet without restriction (regular diet), matching the case group for age and sex, by means of a mathematical random function. In both groups, exclusion criteria were celiac disease, cystic fibrosis, or inflammatory bowel disease. In the control group, individuals who had LI or reported discomfort after ingesting dairy products were also excluded.

### 2.2. Anthropometric Measurements

Both cases and controls underwent a physical examination with anthropometric evaluation and QUS measurement of the bone status. All cases and controls had reached the Tanner pubertal stage 5. A team formed by two pediatricians, one biostatistician, one dietitian, and one medical student went to the school, presented the research project, and asked the students to fill out a dietary record for the week preceding the day of the examination. The QUS apparatus was then taken to the school gymnasium to examine the volunteers. The anthropometric measures were obtained in the morning using a repeated-measure protocol [17]. Height (m) was measured in the upright position to the nearest 1 mm with a standard stadiometer (Bilance Salus, Milano, Italy). Body weight (kg) was measured on a mechanical scale with a precision of 100 g (Bilance Salus, Milano, Italy). Body mass index (BMI) was calculated as body weight divided by height squared (kg/m^2^). All subjects were defined as underweight (under 3rd percentile), normal weight (between 3rd and 85th percentile), overweight (between 85th and 95th percentile), or obese (≥95th percentile) on the basis of the Cacciari’s growth chart percentiles for Italian children and adolescents [18]. The study was approved by the Ethics Committee of the University Hospital S. Anna in Ferrara, Italy (ethical code 120198/8-2012 approved on 26 January 2012). An informed written consent was obtained from the subjects, or the subjects’ parents when under 18, before enrollment.

### 2.3. Diet Record and Ca Intake

The diet record was designed to analyze the diet during one week (7 days) to estimate, in particular, the average daily intake of Ca and vitamin D. The diet record considered consumption (amount and servings/day) of foods that are a source of Ca and vitamin D: milk and dairy products (yogurt, cheese, and butter), water, vegetables, fresh and dry fruit, fish, and sweets and desserts containing dairy products. To define the quantity, the record contained pictures with example of servings from the dietary atlas by Istituto Scotti Bassani, Italy (www.scottibassani.it). Bottled water was classified in three categories depending on the total dissolved solids (TDS) value reported on the bottle label: very low mineral content (TDS < 50 mg/L), low mineral content (50 mg/L < TDS < 500 mg/L), medium mineral content (500 mg/L <TDS < 1500 mg/L). Ferrara’s tap water TDS was provided by the public water company (HERA, Bologna, Italy). The diet record required also information on the weekly hours of physical activity performed: if it was limited to the two hours at school or if the subject practiced extra-curricular activity; how many hours per week were devoted to the extra-activity; the type of exercise, i.e., weight-bearing (e.g., ball games, aerobics, running) or non-weight-bearing (e.g., cycling, swimming). The Population Reference Intake (PRI) of nutrients for the Italian population (LARN) was based on the guidelines issued by the Italian Society of Human Nutrition (www.sinu.it) [19]. At the hospital, the diet record was filled out with the help of the parents. At school, the diet record was provided before our arrival, and the children filled it out at home with the parents. For both sites, the record was checked by the team’s dietitian, and the adolescents were interviewed to ensure the accuracy of the information. The data was analyzed using the software WinFood (version 3.6 Pro, Medimatica, Teramo, Italy). The milligrams of Ca per day are expressed as mean ± standard deviation (SD).

### 2.4. Quantitative Ultrasound

Quantitative ultrasound measurements were obtained using a DBM Sonic Bone Profiler BP01 (Igea Ultrasonics, Carpi, Italy). Ultrasound attenuation of the amplitude-dependent speed of sound (AD-SOS) and bone transmission time (BTT) were measured at the diaphysis of the proximal phalanx of the four fingers of the non-dominant hand [20,21,22]. In a pediatric population, an AD-SOS age-related Z-score value <−2 SD identifies a condition of “low bone mineral status” according to the anthropometric variable considered, as suggested for DXA measurements by the International Society for Clinical Densitometry [23]. Baroncelli et al. [24] have provided, for phalangeal QUS, a large reference database for healthy Italian subjects aged 2–21 years according to the main anthropometric findings, including pubertal stages and body mass index, expressed as percentile. To correct for the thickness of the surrounding soft tissues of the hand, the probe of the phalangeal QUS device was applied also to the soft tissue area. This value was then automatically used by the device when measuring AD-SOS in the phalanx to take account, at least in part, of soft tissue interference [25]. Subjects were grouped into normal and low BMS according to the AD-SOS age-related Z-score value. QUS measurements were made by two experienced operators. The Intraclass Correlation Coefficient (ICC) was calculated as index of inter-rater reliability (0.91). The QUS device was calibrated daily by a standardized phantom.

### 2.5. Breath Test

The breath test had been performed for the cases for diagnostic purposes, as previously reported [26]. In detail, the subjects received an oral load of 1 g of lactose/kg of body weight, up to a maximum of 50 g, dissolved in a water solution. One breath sample was taken before the oral lactose load and every 30 min thereafter for a 3 h period. Hydrogen and methane concentrations were measured in parts per million (ppm) by means of a MicroLyzer gas chromatograph (Model DP, Quintron Instruments, Milwaukee, WI, USA) with a solid-state sensor detector (sensitivity 1 ppm, accuracy 2 ppm, linear range 2–150 ppm). The test was judged to be positive when the peak of hydrogen or methane exceeded 20 ppm over the baseline value.

### 2.6. Statistical Analysis

The Student’s *t*-test was used to analyze differences in quantitative data, and the values were given as mean ± standard deviation. The Shapiro–Wilk test was applied to check for normal distribution, and the Bartlett test to check for equal variances across the samples. When not normally distributed, the values were transformed using the Box–Cox transformation. If it was not possible to normalize the data, they were transformed as close as possible to a normal distribution. The Fisher’s exact test was used to examine the significance of the association in 2 × 2 contingency tables. Generalized linear models were performed to analyze the association of linear or binary outcomes with multiple variables and to adjust for covariates. The Pearson’s correlation coefficient (R) was computed for the parametric estimates of the level of association between the variables. For non-parametric variables, the Spearman’s correlation was used. A statistical analysis was performed with the software R version 3.2.2 (R Foundation for Statistical Computing, Vienna, Austria, www.r-project.org). The following R packages were used: ‘irr’ version 0.84 to calculate the ICC, ‘AID’ version 1.5 for the Box–Cox transformation, and ‘pwr’ version 1.1.1 to check the statistical power based on the sample size, standard deviation, and mean to have type II error (beta) > 80%. A *p*-value < 0.05 (type I error or alpha <0.05) was considered statistically significant.

## 3. Results

### 3.1. Subjects

There was no statistically significant difference between cases and controls in terms of mean age and BMI. When stratifying the two groups by sex, males in the control group had significantly higher values of BMI (23.5 ± 3.3 kg/m^2^ vs. 21.6 ± 3.8 kg/m^2^, *p* = 0.01) and weight (70.9 ± 14.8 kg vs. 64.1 ± 14.1 kg, *p* = 0.01) than males in the case group. No significant difference was observed between female cases and controls (Table 1). Eighteen cases were above the 85th percentile (nine overweight and nine obese). Among controls, 14 were overweight and six obese. Two cases and three controls were underweight. The subjects did not suffer from any fragility fracture at the hands.

### 3.2. Quantitative Ultrasound

The AD-SOS age-related Z-score did not differ between cases and controls (*p* = 0.75). When stratifying by sex, no statistical difference was observed between cases (Female (F): 0.27 ± 1.35 vs. Male (M): 0.21 ± 1.40, *p* = 0.30) and controls (F: 0.29 ± 1.13 vs. M: 0.27 ± 1.22, *p* = 0.92).

The AD-SOS age-related Z-score was inversely correlated to the BMI (*R* = −0.39, *p* = 1.7 × 10^−8^) both in cases and controls (respectively: *R* = −0.35, *p* = 2 × 10^−4^ and *R* = −0.46, *p* = 8 × 10^−6^), also when adjusted for hours spent exercising and Ca intake. Twelve cases (11.8%) had low BMS. In the control group, five subjects (4.9%) had low BMS. The BTT age-related Z-score did not differ between cases and controls (*p* = 0.75). When stratifying by sex, no statistical difference was observed between cases (F: 0.36 ± 0.94 vs. M: 0.01 ± 1.14, *p* = 0.09) and controls (0.38 ± 1.22 vs. 0.15 ± 1.02, *p* = 0.31).

Among the cases, there was no difference between LI and LT subjects in terms of AD-SOS age-related Z-score (*p* = 0.11) and BTT age-related Z-score (*p* = 0.07). No difference was found between males and females, both in cases and controls, when comparing the AD-SOS and BTT age-related Z-score.

At the time of this study, the cases had been on a lactose-free diet for 4.8 ± 3.1 years. The AD-SOS age-related Z-score was inversely correlated to the time spent in the lactose-free diet (*R* = −0.32, *p* = 0.003). To further investigate, the cases who had been for more than 4.8 years on a lactose-free diet (*n* = 40, 23 F and 17 M) were compared to a randomized control subgroup matched for number and age. There was no difference in the AD-SOS age-related Z-score (*p* = 0.13), while there was a difference in Ca intake (766 ± 497 vs. 1077 ± 490 mg/day, *p* = 0.01).

### 3.3. Dietary Intake of Ca and Vitamin D and Correlation to Physical Exercise

The lactose malabsorbers had a lower Ca intake and a lower Vitamin D intake than the control group (Table 2). Control males had a higher Ca intake than females (*p* = 6 × 10^−4^). This difference was not observed in the case group (*p* = 0.86).

**Figure 1:**
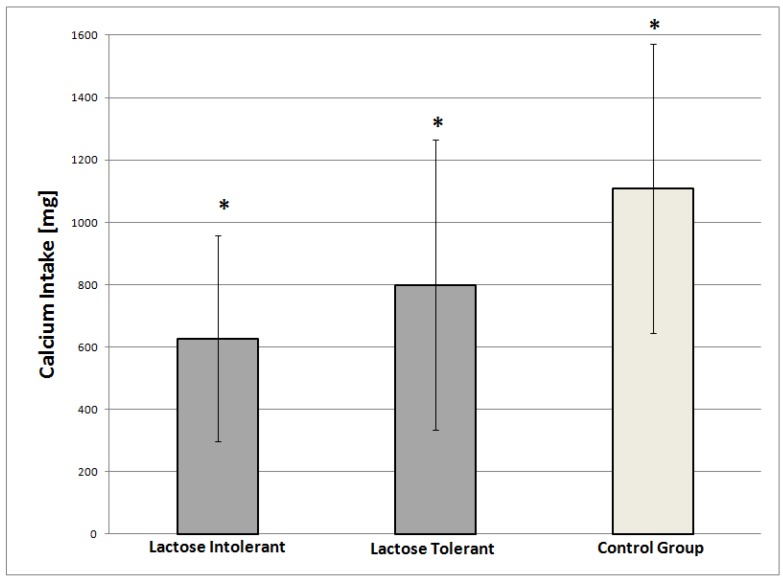
Amount of Ca intake (mg/die) in lactose intolerant (*n* = 41), lactose tolerant (*n* = 61), and control subjects (*n* = 102); *: statistically significant difference between the three groups.

Among the cases, the LI patients had a significantly lower Ca intake than the LT patients (624.8 ± 331.6 vs. 799.6 ± 466.6 mg/day respectively, *p* = 0.03) (Figure 1), with no evident role of a food group over the others.

There was no difference between the two subgroups in terms of Vitamin D intake (*p* = 0.62).

About 75% of cases drank milk (98% of whom drank lactose-free cow’s milk and about 2% soy milk), and nearly 83% of controls drank cow’s milk with the same average frequency (Table 2). There was no difference in milligrams/day of Ca intake through milk between cases and controls (*p* = 0.78). In the control group, males introduced significantly more Ca than females (*p* = 0.001)

Less Ca was significantly introduced by the cases through dairy products (yogurt, butter, soft and aged cheese), fruit (fresh and dry), legumes, fish, and sweets and desserts containing dairy products. 

Parmesan cheese was considered separately from the aged cheese group because of its high Ca (1159 mg Ca/100 gr) and very low lactose content [27]. The cases introduced a significantly lower amount of Ca from Parmesan cheese than the controls. In both groups, the highest percentage of Ca intake was provided by milk (cases: 28.7%, controls: 19.3%), followed by Parmesan (cases: 16.3%, controls: 18.9%).

**Figure 2:**
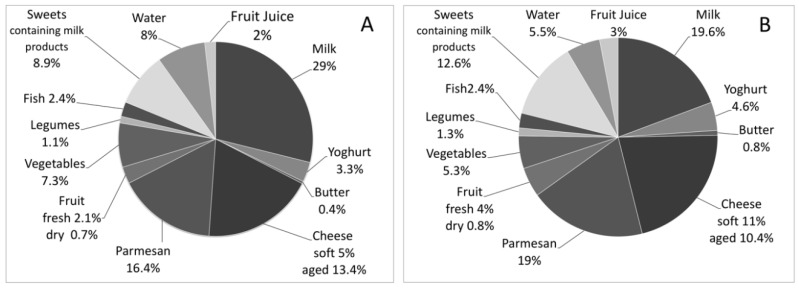
Percentage of Ca intake from different food categories. Ca intake was calculated by analyzing the consumption (amount and times/week) of Ca-rich foods (milk and dairy products, water, vegetables, fresh and dry fruit, legumes, fish, and sweets and desserts containing dairy products). (**A**) Percentage of Ca intake in the cases; (**B**) Percentage of Ca intake in the controls.

Water provided 8.0% of Ca in the cases and 5.5% in the controls, the highest percentage deriving from water with medium mineral content (cases: 44.4%, controls: 21.9%) (Figure 2). There was no significant difference in Ca intake from water between cases and controls (Table 2).

On average, the number of weekly hours spent exercising were 5.2 ± 3.2. The controls spent significantly more time practicing physical activity than the cases (6.1 ± 3.6 vs. 4.3 ± 2.7, *p* = 1 × 10^−4^, respectively). About 73% of the controls and 23% of the cases (of whom, 61% were lactose-tolerant) did extra exercise in addition to the curricular hours at school. During the extra hours, about 98% of the controls and 72% of the cases practiced weight-bearing sports, mostly soccer and basketball. 

A linear correlation was observed between the average Ca intake (mg/day) and the weekly hours spent doing physical exercise (*R* = 0.21, *p* = 0.002). There was no significant correlation between Ca intake and BMI (*p* = 0.44), either in the cases or in the controls. The number of weekly hours spent doing physical exercise did not correlate either with the AD-SOS Z-score (*p* = 0.16) or with the BTT Z-score (*p* = 0.30).

### 3.4. Ca Intake and BMS

No significant association was observed between dietary Ca intake and AD-SOS age-related Z-score (*p* = 0.38) or BTT age-related Z-score (*p* = 0.21). In subjects with low BMS, there was no significant difference between cases and controls in Ca intake (750.4 ± 536.5 vs. 907.8 ± 268.1 mg/day respectively, *p* = 0.45) nor in hours spent exercising (4.2 ± 3.3 vs. 6.2 ± 3.0, *p* = 0.30). Cases and controls with low BMS (*n* = 17) did not significantly differ in Ca intake from those with normal BMS (*n* = 187) (765.4 ± 475.5 vs. 940.9 ± 480.8 mg/day respectively, *p* = 0.08).

## 4. Discussion

Adequate Ca intake during the growth years is considered to be critical for reaching the optimal bone accrual and prevent the risk of osteoporosis in adulthood [7]. For the Italian population, the Ca PRI is 1300 mg/day. Milk and dairy products are the primary source of Ca, but children with adult-type hypolactasia usually need to follow a lactose-free diet to avoid gastrointestinal symptoms. Some of these results were presented at the SIGENP meeting 2018 in a poster session [28].

### 4.1. Ca Intake

Surprisingly, 98% of the cases drank lactose-free cow’s milk. This kind of milk contains less than 0.5% of lactose and has all the properties of regular cow’s milk, including the presence of bioactive components such as the insulin-like growth factor 1 (IGF-1), that may facilitate bone growth [29]. Lactose-free cow’s milk appears to be an alternative to regular milk that allows lactose malabsorbers to introduce a good amount of Ca and bioactive components. Nevertheless, despite the substitution of regular cow’s milk with lactose-free cow’s milk, in our population, the malabsorbers had a Ca intake significantly lower than in the controls. Also, we observed that the cases (lactose malabsorbers) introduced less dairy products (yogurt, butter, and cheese) than the controls. This behavior was primarily linked to the concern for having symptoms.

In addition to milk, water may be another important source of Ca, as the dissolved minerals are highly bioavailable [30]. In our study, the medium mineral content water provided was up to 44% of daily Ca intake. Thus, water can cover a good amount of Ca PRI.

The data showed that Ca intake, as well as the intake of vegetables, legumes, fruit and fish in children and adolescents was lower than recommended, both in cases and in controls.

However, we have to consider that bone development is not only limited to Ca intake. As mentioned previously, cow’s milk provides several nutrients that are important for body and skeletal health [29,31]. Of note, the patients who had had gastrointestinal symptoms during the breath test (LI), introduced significantly less Ca than the patients who had not developed adverse effects after the lactose challenge (LT). We suppose that the first group was not able to tolerate even small amounts of lactose and followed the diet more strictly than the latter group. In primary lactose deficiency, some people can ingest a limited amount of lactose before developing any symptoms [4]. Again, the concern about having symptoms influenced the intake of dairy products.

### 4.2. Phalangeal BMS

In the literature, the majority of data on bone mineralization has been obtained by DXA or quantitative computed tomography (reporting BMD). Recent studies have clarified most of the dubious technical aspects of QUS leading it to its clinical application in a number of disorders [22,32,33]. Normative data has been reported in big cohorts of children and adolescents [24,34,35,36]. QUS diagnostic is a good method to evaluate BMS, as reported by several studies [37,38,39]. We investigated BMS by means of a phalangeal Z-score QUS device to evaluate the effect of the diet on primary-lactase-deficiency adolescents. The analysis of phalanges may serve as a surrogate of the bone quality and structure, but it is limited to the peripheral skeleton [40].

In our study, no significant association was observed between dietary Ca intake and phalangeal BMS, and the BMS was not different in cases and controls, despite the lower Ca intake. These results are in accordance with previous studies [41,42] and in agreement with the findings of a Cochrane review that, in 19 articles including more than 2000 patients, detected only a small and transitory effect of Ca supplementation in healthy children [43]. The BMS is influenced by several factors (e.g., genetic factors, body weight, physical activity), among which, calcium is an important one. The only low calcium intake in healthy adolescents could not be enough to modify the BMS. We think that calcium levels should be further reduced to a threshold that is not reached even in subjects with ATH to be critical for the BMS.

The inverse correlation between the time spent on a lactose-free diet and the AD-SOS age-related Z-score suggests that a lower Ca intake could affect the BMS on a long period of time. Although the difference in BMS was not significant in the sub-groups (LI versus LT), the AD-SOS age-related Z-score was lower than in the controls.

### 4.3. Physical Activity

Physical activity seems to be the primary modifiable stimulus for increased bone growth and development in adolescents [44], accounting for 10–22% of adult bone variance [45]. The reported effect of physical exercise was correlated with the bone mineral density (BMD) obtained by DXA at different sites than phalanges. About 93% of our subjects practiced weight-bearing exercises [32,46]; however, using QUS, we did not observe a correlation between hours of physical activity and AD-SOS age-related Z-score. In this study, QUS is not used as an alternative technique to DXA. The use of QUS is aimed to monitor and then prescribe further and more specific exams.

The controls spent more time doing extra-curricular exercise compared to the cases, who limited their physical activity to the two hours at school time. Other studies observed a correlation between physical exercise and Ca intake [46,47]. They also reported a correlation with BMD. However, those studies used DXA and examined different body sites. Probably, physical exercise might not significantly influence the BMS of the phalanges, as they are not a weight-bearing site and are thus less affected by physical exercise than other sites.

## 5. Conclusions

The importance of cow’s milk and Ca intake in adolescents is still controversial. This study showed that lactose malabsorbers had a good Ca intake thanks to lactose-free cow’s milk, and a Z-score comparable to the controls.

Our results indicate that a lactose-free diet does not affect the phalangeal BMS of adolescents with primary lactase deficiency when their diet includes lactose-free cow’s milk. However, these patients still have a significantly lower intake of Ca compared to peers on a regular diet. The inverse correlation observed between the AD-SOS age-related Z-score and the time spent on a lactose-free diet suggests that a long-term follow up of the malabsorbers is advisable.

## Figures and Tables

**Table 1:** Characteristics of the study subjects.

	Cases		Controls		
		*n*		*n*	*p*-Value
Age (years ± SD)	16.8 ± 2.5	102	17.0 ± 2.2	102	
M	16.7 ± 2.4	45	17.2 ± 2.0	48	
F	16.9 ± 2.6	57	16.8 ± 2.0	54	
BMI (kg/m^2^)	21.5 ± 3.6		22.4 ± 3.4		0.08
M	21.6 ± 3.8	45	23.5 ± 3.3	48	0.01
F	21.5 ± 3.5	57	21.5 ± 3.2	54	0.98
Weight (Kg)	60.0 ± 12.4		63.0 ± 14.2		0.1
M	64.1 ± 14.1	45	70.9 ± 14.8	48	0.01
F	56.7 ± 9.8	57	56.3 ± 9.5	54	0.92
Height (m)	1.66 ± 0.09		1.67 ± 0.09		0.21
M	1.71 ± 0.09	45	1.74 ± 0.08	48	0.1
F	1.62 ± 0.05	57	1.62 ± 0.06	54	0.7

BMI: body mass index; M: male; F: female; SD: standard deviation.

**Table 2:** Comparative analysis of the Ca intake divided into food categories.

	Cases			Controls			
	Ca Intake (mg/day)	Frequency	*n*	Ca Intake (mg/day)	Frequency	*n*	*p*-Value
	(Mean ± sd)	(Days/Week)		(Mean ± sd)	(Days/Week)		
**Total Ca intake**	**752.1 ± 433.4**		102	**1108.6 ± 463.8**		102	7.4 × 10^−8^
M	763.4 ± 424.0		45	1289.3 ± 487.3		48	6.4 × 10^−7^
F	743.3 ± 444.3		57	964.7 ± 392.3		54	0.006
Milk	216.4 ± 256.2	4.5 ± 2.8	76	214.6 ± 181.0	4.6 ± 2.3	83	0.78
Yoghurt	25.2 ± 54.6	2.8 ± 2.3	48	51.3 ± 41.7	2.4 ± 1.7	67	3.4 × 10^−7^
Butter	2.9 ± 8.0	1.4 ± 1.2	54	9.2 ± 13.8	1.8 ± 1.3	67	7.3 × 10^−6^
Cheese							
soft cheese	38.2 ± 55.8	2.0 ± 1.5	79	122.0 ± 76.4	3.3 ± 1.6	94	4.0 × 10^−15^
aged cheese	101.0 ± 124.5	1.9 ± 1.6	68	115.1 ± 117.4	1.9 ± 1.5	80	0.0009
Parmesan	123.2 ± 112.2	4.9 ± 2.4	58	210.5 ± 141.0	5.1 ± 2.7	77	0.0001
Fruit							
fresh	15.8 ± 40.1	5.9 ± 1.9	34	43.4 ± 34.5	5.4 ± 1.9	91	7.7 × 10^−15^
dried	5.5 ± 9.6	1.9 ± 2.2	40	9.0 ± 7.3	2.0 ± 1.5	54	0.008
Vegetables	55.1 ± 64.0	5.4 ± 2.2	40	58.2 ± 39.6	5.7 ± 1.8	95	0.71
Legumes	8.6 ± 8.0	1.5 ± 1.0	66	14.3 ± 24.3	1.8 ± 1.1	78	0.051
Fish	18.2 ± 26.2	1.3 ± 0.8	83	26.4 ± 33.8	1.8 ± 1.1	88	0.025
Sweets containing milk products							
cookies and snacks	28.4 ± 18.5	5.0 ± 2.5	79	37.2 ± 33.8	4.4 ± 2.3	87	0.025
Ice-cream and pudding	39.3 ± 63.2	3.1 ± 2.4	67	103.1 ± 101.2	3.0 ± 2.0	87	3.9 × 10^−7^
Water	60.7 ± 97.6		102	61.4 ± 101.0		102	0.19
very low mineral content	15.7 ± 8.4		10	27.3 ± 17.9		6	0.12
low mineral content	48.0 ± 27.2		83	44.2 ± 24.9		87	0.15
medium mineral content	334.6 ± 160.0		9	243.7 ± 274.2		9	0.34
Fruit Juice	13.8 ± 5.6	5.0 ± 2.0	16	33.0 ± 24.7	4.3 ± 2.5	58	4.7 × 10^−6^
Carbonated drinks			31			52	
**Total Vitamin D intake**	Vitamin D intake (µg/day) (mean ± sd)		*n*	Vitamin D intake (µg/day) (mean ± sd)		*n*	*p*-value
	3.6 ± 3.1		102	4.7 ± 2.7		102	0.005
